# The National Hospital for the Paralysed and Epileptic

**Published:** 1900-08-11

**Authors:** 


### The Institutional Workshop.

### THE NATIONAL HOSPITAL FOR THE PARALYSED AND EPILEPTIC.

### CAUSES OF DIFFERENCE.

The differences which have arisen between the honorary medical staff and the board of management of the National Hospital for the Paralysed and Epileptic, Queen Square, will be brought to a head at a special meeting of the governors, to be held on the 11th instant at noon. The issues of the dispute are not understood by the majority of those interested in hospitals, and must therefore be largely confusing and unintelligible to the general public. In order to make them as clear as possible we have been at some pains to obtain an impartial view of the case presented by (1) the honorary medical staff; (2) the board of management and the secretary-director. We will first set out

### The Case of the Honorary Medical Staff.

The crux of the whole matter so far as the staff is concerned appears to be their objection to the authority vested in the secretary-director, Mr. Burford Rawlings. They maintain, "the relations between the medical staff and the board of management have shown increasing signs of strain for several years past in proportion as the board has more and more relegated its functions and authority to the hands of a paid official, Mr. Burford Rawlings, who, from the position which he originally occupied as secretary, has been advanced to the dignity of secretary and general director. Those practically acquainted with hospital affairs will scarcely need to be told that the effect of such an appointment is that, although the management of the hospital may be nominally directed by a board, the institution is practically, to a great and dangerous extent, in the hands of one man. That the autocracy of the office referred to should be as complete as possible it is enacted in the rules of the hospital that no member of the medical staff shall be allowed to have a seat on the board. The secretary and general director is thus relieved of any inconvenience which might possibly be occasioned by the presence on the board of persons professionally acquainted with the work and necessities of a medical institution and able to supply essential opinion and counsel. It is not surprising, therefore, that for a number of years the medical staff have found that the administration of the hospital was becoming more and more unsatisfactory." Various plans emanating from the board appear to have been tried to meet the wishes of the staff in regard to representation. A plan of sending delegates, who should be summoned to attend the board whenever matters affecting the medical staff were under discussion, was given a trial, but it was repudiated by the medical staff in consequence of the delegates not being summoned by the board upon one occasion, an omission which the medical staff attribute to the bad faith of the board, though the board expressed their regret that this error was committed, and they undertook that it should not be repeated. The particular meeting to which the medical delegates were not summoned appears to have been one when the board determined to introduce a system of payment by out-patients, to which the medical staff have a deep-rooted objection. The medical staff maintain, further, that the Hospital Sunday Fund Distribution Committee on July 13th, 1899, instructed the secretary-director to inform his board that the medical staff must be directly represented on the board of management or the hospital would not receive any grant of money in 1900. The board reply that the recollection of the secretary-director is that the Hospital Sunday Fund Committee did not express any opinion that the medical staff should be directly represented on the board, and the statement of Sir Sydney Waterlow at the Mansion House on July 30th to the effect that the Distribution Committee felt that this was a question to be settled not by the Hospital Sunday Fund but by the hospital itself, "by the parties to the dispute and the governors and subscribers," would seem to confirm the impression of the board on this point. Sir Sydney distinctly laid it down that it was not the duty of the Committee of Distribution to interfere with the inside management of hospitals receiving grants from the Hospital Sunday Fund. In any case, no written communication on the point appears to have been received by the board during 1899 from the Hospital Sunday Fund authorities.

The medical staff maintain that the patients' diet is often deficient in quantity and quality, and that, in consequence, patients' friends are distinctly encouraged to bring the in-patients articles of food from outside by a notice posted in the hospital sanctioning the introduction of certain kinds of food. The staff maintain that patients are often not kept clean as regards their bodies; that sufficient clean linen is not supplied; that the treatment ordered is not efficiently carried out; that the washing of draw sheets is neglected; and that adequate provision for washing enough bed-linen has been refused by the management; that the nursing staff is deficient in numbers, there being no reserve of nurses for an adequate supply of substitutes; that the accommodation for nurses is insufficient, and the food supplied to them exceedingly defective in quality. The medical staff further maintain that the board sanction the taking of money from patients in exchange for their out-patient letters, a practice which, though stopped in 1887 on representations from the medical staff, was renewed in 1891, when they again called attention to the matter and were censured by the board for so doing.

### The Case of the Board.

The board maintain that the several charges affecting the well-being of the patients came to their knowledge for the first time by the issue of the medical staff's statement to the governors, issued so recently as the middle of June, 1900, of which no copy was sent by the medical staff to the board. A sub-committee of the board, consisting of Mr. G. W. E. Russell, chairman; Sir Gerald Fitzgerald, K.C.M.G., and Mr. John Pearman, has investigated (1) the alleged insufficiency in the quantity and quality of the diets supplied to the patients; (2) the condition, care, and treatment of the patients; and (3) the nursing of the hospital. They state with regard to all the charges that "the medical staff have never brought any of them to the attention of the secretary-director or of the board, as it was their bounden duty to do if they considered they were well founded. In the course of their investigations they paid a surprise visit to the kitchens, and also to some of the wards where dinner was being served." They examined the various officers, including the secretary-director, the chaplain, the senior house physician, the lady superintendent, the present and late assistant matrons, and the whole of the sisters of the hospital, together with the male staff nurses, the steward, and the cook. "After careful and exhaustive examination of the witnesses, many of whom were able to speak from a lengthened experience of the working of the hospital, the Committee of Enquiry are of opinion that the charges made by the medical staff are not justified." The committee in their report take each of the three foregoing matters separately, and deal with it fully and in detail. We have no space to reproduce the report, but a copy of it will no doubt be sent to any one interested, on application to the secretary-director at the hospital.

On the charge of maladministration of the hospital, the board in their report state that "it will be noted that the medical staff write of maladministration as though it were proved, but the board feel confident that the governors will require something more than bare assertions, unsupported by evidence, before proceeding to condemn an administration which has been attended with results uniformly successful." The board admit the eminence and brilliance of the medical staff, "which have always been a source of pride to the hospital," but they point out that "however eminent and brilliant the medical staff may be they rest under the ordinary obligation to make good their assertions by proof, which they have failed to do." The board emphatically deny "that the existence of the office of general director has rendered them less accessible to the staff, or less mindful of the duties pertaining to their office. The present holder of the office, Mr. B. Burford Rawlings, was associated with the founders of the hospital. He has seen every member of the staff but one enter the service of the hospital. In view of the attack now made on Mr. Burford Rawlings, the board deem it a simple act of justice to record that for the past thirty-four years he has devoted his time and talents to the hospital, and that his efforts have been attended with conspicuous success. He has initiated every enlargement and identified himself with every feature of its life. He successfully carried through the rebuilding of the hospital, and subsequently of the convalescent home, at a cost of £115,000, without remuneration; the amount collected by his letters, personal influence, and writings forming a very large proportion of that total, while his personal collections on behalf of the hospital reached nearly £150,000, all be it remarked outside of what is required of him by the rules. Mr. Burford Rawlings enjoys the deserved confidence of the board, and he has informed the board that down to a very recent period his relations with the members of the medical staff were, with a single exception, friendly, and in most cases cordial. The best evidence of the success which has attended his administration of the hospital is that which is afforded by an inspection of the institution and by inquiry into its working and results." The board intimate that they would gladly welcome the most searching investigation, and feel confident that the result would be the triumphant vindication of the hospital and its management.

The objections of the board to the medical staff being directly represented on the Committee of Management may be summarised as follows: "These objections are summed up in the proposition that members of the medical and surgical staff, being officers and servants of the hospital, should not be included in the governing body. The board maintain (1) that as the members of the medical staff are charged with the performance of definite duties, questions referring to their discharge of those duties may arise at any time; (2) in the company of members of the medical staff, the independence of the board during discussion would disappear, for while the Medical Committee would continue to meet alone the board's deliberations would be always shared by the medical staff; (3) presence upon the board would involve presence upon the House Committee, where complaints by patients sometimes in reference to actual or so-called neglect by medical officers are heard. The interference of the medical staff would show itself in every detail of management and finance, and instead of being maintained for the benefit of the poor and suffering, the hospital would be in danger of having the philanthropic aspect of its work subordinated to that which is merely scientific and investigatory. The claims of mere suffering would be likely to be overlooked, and the disposition, generally present, to give preference to 'cases' introduced by fellow-practitioners, while patients recommended by subscribers are kept waiting, would be aggravated. Already nominations for admission to the hospital are very largely monopolised by the medical staff, their friends, and their clients. The medical members, though only two in number, would be delegates having the force of the whole Medical Committee behind them, and would thus be in a position to enforce their views by threatening hostilities on the part of the Medical Committee, if the lay members opposed them. By force of sheer obstinacy in the maintenance of firmly held views, and animated by the unyielding conviction that they were better informed than their lay colleagues, the two medical members would in time dominate the whole proceedings of the board." Finally, "the undue exaltation of medical authority would mean that the matron, sisters, and nurses would learn to look to the Medical Committee, from which they are now, and with great advantage, wholly separated. At present the secretary and director is the officer and servant of the board, and his independence of the Medical Committee is absolutely a fact to which the board attach the greatest importance. To include members of the medical staff on the board would alter his relations to the medical staff, and render some of his most useful functions almost impossible of performance."

The foregoing is but a rough summary of the main matters in dispute stated from the point of view of both sides. We have made a most careful and complete inspection of the hospital since the various parties have issued their manifestoes, and have satisfied ourselves that there are few, if any, hospitals in the metropolis as efficient as the National Hospital for the Paralysed and Epileptic undoubtedly is at the present time. The patients appear to be contented and well cared for; the hygiene and general up-keep of the hospital is good and well maintained; there is an atmosphere of sound ventilation and solid comfort; everything is beautifully clean; and any visitor possessed of a good knowledge of hospital administration must be charmed with most of the arrangements and the general appearance of the wards and appliances, as well as of the patients and the nursing staff. The nursing staff are well satisfied and most loyal to their institution, as we were at pains to ascertain. Pending the meeting of the governors we desire to express no final judgment on the matters in dispute, but we may properly state that, after our inspection, as the result of great experience, we are at a loss to understand how many of the charges made can have been formulated by eminent members of the medical profession in regard to so efficient and well-administered a hospital.

On the 8th inst. we received a copy of the letter from the Honorary Medical Staff of the National Hospital for the Paralysed and Epileptic which was published in several journals last week. The original date of the letter has been altered from July 27th to August 7th, but with that exception the letters appear to be identical. We have already quoted from this letter in the preceding article, and had a copy been sent to us we should have been glad to have published it in full last week in the ordinary course.

